# OCCURRENCE OF UNUSUAL HAEMOGLOBINOPATHIES IN BALOCHISTAN: HB SD AND HB SE - PRESENTATION WITH OSTEOMYELITIS

**DOI:** 10.1590/1984-0462/2021/39/2019365

**Published:** 2021-02-03

**Authors:** Usman Tauseef, Misbah Anjum, Mohsina Ibrahim, Hina Sabih Baqai, Abubakar Tauseef, Marium Tauseef, Muhammad Sohaib Asghar, Maryam Zafar, Uzma Rasheed, Nimra Shaikh

**Affiliations:** a National Institute of Child Health, Karachi, Pakistan.; b Dow University of Health Sciences, Karachi, Pakistan.; c Jinnah Sindh Medical University, Karachi, Pakistan.; d Liaquat National Hospital and Medical College, Karachi, Pakistan.

**Keywords:** Hemoglobin, Sickle cell anemia, Electrophoresis, Anemia, Osteomyelitis, Hemoglobina, Anemia falciforme, Eletroforese, Anemia, Osteomielite

## Abstract

**Objective:**

To describe two cases of unusual variants of sickle cell disease.

**Case description:**

We present two cases of sickle cell disease variants (haemoglobinopathies), from unrelated families, in the state of Balochistan (Pakistan). One was diagnosed with sickle cell disease in the haemoglobin electrophoresis, whereas the other was diagnosed with sickle cell SE disease. Both were diagnosed based on the presentation of osteomyelitis.

**Comments:**

Haemoglobin SD disease (Hb SD) and haemoglobin SE disease (Hb SE) are rare haemoglobinopathies in the world. The lack of available literature suggests that both are variants of sickle cell disease (SCD), with heterogeneous nature. The prevalence of sickle cell disease with compound heterozygotes was found at a variable frequency in the population of the Asian Southeast. The frequency of osteomyelitis in SCD is 12 to 18%, but its occurrence among variant haemoglobinopathies is little reported. Both reported cases presented with osteomyelitis as a characteristic of the disease presentation.

## RESUMO

**Objetivo:**

Descrever dois casos de variantes raras da hemoglobinopatia falciforme.

**Descrição do caso:**

Apresentamos aqui dois casos de hemoglobinopatias variantes das células falciformes, de famílias não relacionadas, no estado do Baluchistão (Paquistão), sendo um diagnosticado como doença da hemoglobina SD na eletroforese de hemoglobina, enquanto o outro com doença da hemoglobina SE. Ambos foram diagnosticados a partir da apresentação de osteomielite.

**Comentários:**

Hemoglobina SD (Hb SD) e hemoglobina SE (Hb SE) são hemoglobinopatias raras no mundo. A escassez de literatura disponível sugere que ambas são variantes da doença falciforme (DF) com natureza heterogênea. A prevalência de hemoglobinopatia falciforme com heterozigosidade composta foi encontrada com frequência variável na população do sudeste asiático. A frequência de osteomielite na DF é de 12 a 18%, mas sua ocorrência entre as hemoglobinopatias falciformes variantes é pouco relatada. Os dois casos reportados apresentaram osteomielite como característica de apresentação da doença.

## INTRODUCTION

Haemoglobin SD disease (Hb SD) and haemoglobin SE disease (Hb SE) are both variants of sickle cell disease (SCD), which is a defect of a beta-globin chain of haemoglobin leading to polymerization of haemoglobin resulting in a vaso-occlusive crisis and other clinical manifestations.^1^ Hb SD and Hb SE are deviant haemoglobins with mutations affecting the beta-globin gene.^2^ All hereditary haemoglobinopathies are distributed worldwide depending upon the variants. Both Hb SD and Hb SE can be clinically silent with characteristic laboratory outcomes of declining haematological indices. The definitive diagnosis is made on the results of haemoglobin electrophoresis.

Osteomyelitis is defined as an infection of the bone, that has risk of occurrence in patients with SDC secondary to abnormal haemoglobin, that causes decreased vascularity in small vessels of bones. Osteonecrosis and septic arthritis along with osteomyelitis are the most common musculoskeletal manifestations of SDC, due to the vaso-occlusive phenomenon.^3^

We hereby present two rare cases of sickle cell variants, presented to us with osteomyelitis, which is traditionally supposed to be a rare feature amongst the sickle cell variants due to fewer chances of developing vaso-occlusive crisis. Hence, the objective of this study was to recommend the screening of paediatric population diagnosed with osteomyelitis, in order to rule out sickle cell gene abnormalities and, further, to manage them accordingly.

## CASE 1

A 10-years-old boy, resident of city Panjgur, the state of Balochistan, presented in an outpatient clinic with complaints of pain in the left thigh for 15 days along with fever and difficulty in walking for seven days. His past history was unremarkable, except that the child had a history of recurrent episodes of lower limb pain since the age of four years, for which no specific workup or treatment was done. Transfusion history was also negative. He was born to non-consanguineous parents whose family history was negative for haemoglobinopathies or blood transfusions. On physical examination, child was conscious and oriented with no dysmorphic features; he was febrile at the time of examination, with a temperature of 38.3°C, and he was mildly anaemic. Anthropometric measurements were lying at the 5^th^ percentile on the growth chart developed by National Centers for Health Statistics - CDC for age and gender. There was diffuse swelling over the anteromedial surface of the middle third part of the left thigh. Skin overlying swelling was warm with no overlying colour changes. The area was tender on palpation and passive and active movements. The examination of both hip and knee joints was unremarkable. On abdominal examination, the liver was palpable 2 cm below the right costal margin with a total span of 12 cm and the spleen was not palpable. His rest of the systemic examination, including the cardiovascular, respiratory, central nervous system, and musculoskeletal, was also unremarkable.

**Figure 1:**
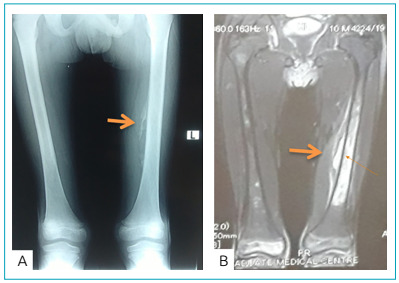
Radiological imaging depicting signs of osteomyelitis. (A) Plain radiograph of left leg indicates mild periosteal reaction and periosteal elevation with sub-periosteal collection and displacement and blurring of fat planes. (B) Magnetic resonance imaging with contrast showing marrow signal abnormality in the mid-shaft diaphysis of the left femur.

Plain radiograph of left leg indicates mild periosteal reaction and periosteal elevation with sub-periosteal collection and displacement and blurring of fat planes, suggestive of infective/inflammatory process (Figure 1A). Magnetic resonance imaging (MRI) with contrast shows marrow signal abnormality in the mid-shaft diaphysis of the left femur with post-contrast peripheral soft tissue signal abnormality and abscess formation in soft tissues, consistent with osteomyelitis (Figure 1B).

His complete blood count shows haemoglobin 6.8 ­g/­dL, haematocrit 21.7%, total leucocyte count 13.5 cells per microliter, comprising of 70% neutrophils and 22% lymphocytes, platelet count of 111,000 per microliter of blood, red cell indices: mean corpuscular volume (MCV) 80.5 fL, mean corpuscular haemoglobin concentration (MCHC) 37.5 ­g/­dL, mean corpuscular haemoglobin (MCH) 30.2 pg, reticulocyte count 7.5% (corrected reticulocyte count 5.0%). Peripheral smear showed anisocytosis, poikilocytosis, target cells, and rouleaux formation. Erythrocyte sedimentation rate of 80 mm/hour (<5 normal) and blood culture revealing no bacterial growth. Haemoglobin electrophoresis result was positive for compound heterozygote for sickle cell SD disease (Table 1).

**Table 1:** Haemoglobin electrophoresis of the patient 1.

Type of haemoglobin	Percentage	Normal range
Hb A2	1.5%	2.4-3.2%
Hb F	15.5%	0.0
Hb D	52.2%	0.0
Hb S	30.8%	0.0
Interpretation	F+S+D+A2	
Compound heterozygote for sickle cell SD		
Methodology: Haemoglobin quantification performed by high-performance liquid chromatography.		

Diagnosis of osteomyelitis of left femur secondary to compound heterozygous SDC (Hb SD) was made, and the patient was given a course of intravenous (IV) antibiotics and hydration. The patient was started on hydroxyurea once the osteomyelitis got resolved, and follow up was planned in the ambulatory clinic of paediatric haematologist after two weeks. The patient’s family was counselled for the screening of the whole family for haemoglobinopathy. The patient’s follow-up remained uneventful.

## CASE 2

A 10-years-old girl, weighing 19 kg, resident of Othal city, State of Balochistan, presented in an outpatient clinic with complaints of fever and pain in the left leg for 10 days. Her past medical history was unremarkable, with no traveling and transfusion history. She was born to consanguineous parents whose family history was negative for haemoglobinopathies. On physical examination, the child was active and alert, sitting on a bed with no dysmorphic features; she was febrile, with 37.7°C, and she was mildly pale and icteric. Abdominal examination revealed soft, distended, tender left hypochondrium with spleen palpable 6 cm below left costal margin while rest of the systemic and local examination was unremarkable, except for some restriction of movements at left hip joint.

Her complete blood count shows haemoglobin 4.9 g/dL, haematocrit 17.5%, total leucocyte count 5.1 cells per microliter with 46% neutrophils and 49% lymphocytes, platelet count of 157,000 per microliter of blood. Reticulocyte count was 5.0%, and red cell indices: MCV 74 fL, MCHC 38.9 ­g/­dL, and MCH 28.8 pg. Peripheral smear showed anisocytosis, poikilocytosis, and target cells. Erythrocyte sedimentation rate was 76 mm/hour (<5 normal), and blood culture revealed no bacterial growth.

**Figure 2:**
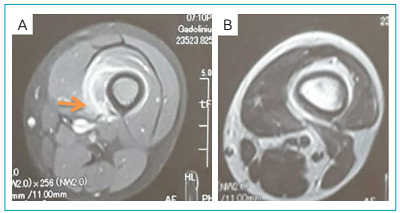
Magnetic resonance imaging showing signs of osteomyelitis. (A and B): MRI with contrast shows signal enhancement of the left femur with soft tissue signal abnormality, suggestive of osteomyelitis.

Bone scan shows areas of increased tracer uptake involving left iliac bone and the distal end of left femur, non-homogenous tracer noted over the dorso-lumbar spine, the rest of the skeleton showed bilaterally symmetrical uptake in the axial and appendicular skeleton. MRI with contrast shows signal enhancement of the left femur with soft tissue signal abnormality, suggestive of osteomyelitis (Figure 2). Haemoglobin electrophoresis result was positive for compound heterozygote for sickle cell SE disease (Table 2).

**Table 2:** Haemoglobin electrophoresis of the patient 2.

Type of haemoglobin	Percentage	Normal range
Hb A1	16.1%	95-98%
Hb F	9.4%	0.0
Hb E	11.5%	0.0
Hb S	63%	0.0
Interpretation	S+E+F+A1	
Compound heterozygote for sickle cell SE		
Methodology: Haemoglobin quantification performed by high-performance liquid chromatography.		

Diagnosis of osteomyelitis of left ilium and femur, secondary to compound heterozygous SDC (Hb SE) was made, and the patient was given a course of IV antibiotics and hydration and oral hydroxyurea. The patient improved with treatment and was able to mobilize independently, so she was discharged on hydroxyurea and was called to follow up after two weeks. Screening of the whole family for haemoglobinopathy was hence advised. The patient was kept under follow-up for further uneventful six weeks with complete resolution of symptoms.

## DISCUSSION

History of haemoglobinopathies is enriched with very unique and uncommon phenomenon, two of them being very peculiar and rare inherited disorders named Hb SD and Hb SE.^1^ ^,^ ^2^ Hb SD and Hb SE are both variants of SDC, which is due to defect in the beta-globin chain of haemoglobin, leading to polymerization of haemoglobin on occasions when it is not bound to oxygen. This polymerization of the beta-globin chain leads to a vaso-occlusive crisis ultimately leading to a variety of symptoms including recurrent pain, dactylitis, acute chest syndrome, autosplenectomy, and osteomyelitis.^3^ SDC is divided into different categories based on their genetic origin and geographical regions, like Central African Republic (CAR), Benin (BEN), Senegal (SEN), Cameroon (CAM), Arab/Indian (ARAB) and Saudi Arabia (KSA).^4^ It is known that the greater the presence of Hb fetal (F) and lesser the presence of Hb sickle (S) in a patient, the ratio of clinical manifestations declines. This is a strong reason for the Arab population bearing less intense symptoms of the disease. SDC is not a defect that purely relies on genetic factors, environmental factors also play a key role in its presentation.^4^ The extremity of weather conditions could be associated with increased frequency of vaso-occlusive crisis, progressing towards frequent hospitalizations among the patient of Hb SD disease.^4^ Along with weather changes, exercise and physical exertion are counted as triggering factors provoking episodes of vaso-occlusive crisis due to metabolic changes such as hypoxia, lactic acidosis, and dehydration.^5^ ^,^ ^6^

Hb SD and Hb SE are deviant haemoglobins with mutations affecting the beta-globin gene.^1^ ^,^ ^2^ Among all types of haemoglobinopathies, Itano, in 1950, identified haemoglobin D as a new variant,^7^ but it was first documented by Vella and Lehman in 1974.^8^ Hb SE disease, in turn, was unearthed in a 70-year aged woman along with her 30-year-old son, natives of Southern Turkey in 1957.^9^ All hereditary haemoglobinopathies are distributed worldwide depending upon the variants, like thalassemia, being prevalent in the Mediterranean region and South East Asia, Hb S prevalent in tropical Africa and among the black population in the new world. Hb D disease is more common among people of India, Pakistan, and Iran.^10^ Northern Europeans are the only population in the world among whom the frequency of Hb SD disease is minimal.^11^ Contrastingly, Hb E is the third most prevalent variant, suspected to affect approximately 30 million people, 80% from South East Asia.^12^ Hemoglobin E (Hb E) is an abnormal hemoglobin with a single point mutation in the β chain. At position 26, there is a change in the amino acid, from glutamic acid to lysine. Hemoglobin E disease (EE) is hemoglobin E disease results when the offspring inherits the gene from both parents. Hb E trait and Hb EE are the mild forms of the disorder. The combination of Hb E and Hb S results in a compound heterozygosity state of Hb SE disease. Hb E has a frequency of about 30-40% in some areas of Thailand, Cambodia, and Laos.^2^ The occurrence of Hb E is also high in India, Nepal, Bangladesh, Pakistan, Vietnam, and Malaysia.^2^ ^,^ ^12^

Hb SD has its mutation usually present on the 121^st^ position of the beta-globin chain in which normal amino acid glutamic acid is replaced by glutamine investigated by haemoglobin electrophoresis.^10^ On the other hand, the patient pronounced as a sufferer of Hb SE disease manifested commonly with substitution of amino acid lysine for the amino acid glutamic acid in position 26 of beta-globin chain detected by haemoglobin electrophoresis.^13^ Haemoglobin D has diverse variants named according the specific state or country in which it is reported; it includes Hb SD Punjab with mutation of the specific kind seen on the 122^nd^ position of beta-globin chain, making it more prevalent and sorted sub-type worldwide.^14^ ^,^ ^15^ Another variant called Hb D disease is reported in Iran, with similar mutation seen on the 22^nd^ position of beta-globin chain, being most prevalent in the Middle East.^16^ ^,^ ^17^ ^,^ ^18^ Homozygous Hb D disease is clinically asymptomatic, it rarely occurs alone in any patient, while double heterozygosity for haemoglobin D with haemoglobin S (HB S) and beta-thalassemia has a variable phenotype depending on the interaction of Hb D with other haemoglobinopathy genes,^18^ ^,^ ^19^ ^,^ ^20^ as was present in our first case.

Hb SE disease presents in the form of heterozygous E trait, homozygous EE disorder and compound heterozygous E along with other unusual haemoglobinopathies and beta-thalassemia variants.^17^ Hb SD and Hb SE, both variants of SDC, are clinically silent, thus minimizing the chances of their diagnosis. So, they are identified occasionally during the investigation of another ailment.^9^ ^,^ ^12^ Hb SD and Hb SE disorders present with prodromal features of moderate normocytic anaemia and splenomegaly.^21^ ^,^ ^22^ In Hb SD and Hb SE disease, we can see target cells, microcytosis, sickle cells, anisocytosis, poikilocytosis, rouleaux formation and polychromatophilia in the peripheral smear. Further clinical presentations recorded are of physical debilitation and gastric irritation.^22^ Patients with Hb SD and Hb SE develop mild unconjugated bilirubinaemia, elevated serum lactate dehydrogenase levels, and hypohaptoglobinemia with normal reticulocyte count.^23^ People with Hb SD or Hb SE disorder are predisposed to bacterial infections, fundamentally those induced by *Streptococcus pneumoniae*, leading to pneumococcal septicaemia.^24^

Laboratory outcomes of both Hb SD and Hb SE are characteristic of declined values of haematological indices that are red blood cells (RBC), haematocrit levels (HCT), MCV, and MCHC.^2^ ^,^ ^21^ Peripheral blood smears of Hb SD and Hb SE depicted the occurrences of target cells, microcytosis, sickle cells, anisocytosis, poikilocytosis, rouleaux formation, and polychromatophilia and no Howell-Jolly bodies were observed.^22^ ^,^ ^24^ Although Hb SD and Hb SE are clinically reserved diseases, but due to polymerization of haemoglobin bonded with oxygen, a number of significant complications have been proclaimed, including chronic haemolytic anaemia interconnected to pulmonary hypertension, bouts of painful ordeals, avascular necrosis of hip and shoulder, acute chest syndrome, sickle cell retinopathy, bone marrow necrosis, haematuria, splenic infarction, splenic sequestration, enlarged spleen, and cholelithiasis.^13^ ^,^ ^21^ ^-^ ^22^

Defined treatment modalities to treat Hb SD and Hb SE disorders are the administration of vaccines against *Pneumococcus* and *Haemophilus influenzae* infections during the neonatal period.^21^ ^,^ ^25^ Hydroxyurea is currently the drug of choice for the treatment of SDC, being recommended for Hb SD and Hb SE.^25^ Supportive measures to be implemented in the case of Hb SD and Hb SE are the utilization of oral rehydration solution, drinking fluids, and avoidance of harsh climatic changes.^21^ For patients of Hb SD and Hb SE undergoing surgical events, relevant safety measures need to be taken during pre-operative, intra-operative and post-operative periods for protection against sickling associated complications like acute chest syndrome.^25^ Transfusing blood products in patients of Hb SE with near to normal haemoglobin ranges would culminate in an unforeseen rise of haematocrit and blood viscosity.^25^

Osteomyelitis is one of the most common musculoskeletal features of the major sickle cell haemoglobinopathies. Its prevalence amongst the patients of SDC is reported to be around 12-18%. Although there is a paucity of data available in the literature involving its presentation in variant sickle cell haemoglobinopathies, the overall risk of osteomyelitis is much higher in beta-globin gene mutations as compared to the general population, owing to the vaso-occlusive crisis. The most common offending agent is *Salmonella* species followed by *Staphylococcal aureus,* but geographical variations do exist.^3^ The diagnostic modality of choice is magnetic resonance scan, and the most common site of involvement is diaphysis of long bones.

In conclusion, these are very rare cases of Hb SD and Hb SE, first-ever being diagnosed in the State of Balochistan, presented to us at the National Institute of Child’s Health (NICH), Karachi. Also, the unique thing about our cases was that both were diagnosed with the presentation of osteomyelitis, which is generally known to occur with vaso-occlusive phenomenon of SDC. However, the variants described in this report are known to have fewer chances of developing vaso-occlusive manifestations. Hence, it is recommended that, in any paediatric case of osteomyelitis, sickle cell gene abnormalities should be screened and ruled out.
